# The art of vector engineering: towards the construction of next‐generation genetic tools

**DOI:** 10.1111/1751-7915.13318

**Published:** 2018-09-26

**Authors:** Luísa Czamanski Nora, Cauã Antunes Westmann, Leonardo Martins‐Santana, Luana de Fátima Alves, Lummy Maria Oliveira Monteiro, María‐Eugenia Guazzaroni, Rafael Silva‐Rocha

**Affiliations:** ^1^ Ribeirão Preto Medical School University of São Paulo Ribeirão Preto, São Paulo 14049‐900 Brazil; ^2^ School of Philosophy, Science and Letters of Ribeirão Preto University of São Paulo Ribeirão Preto, São Paulo 14049‐900 Brazil

## Abstract

When recombinant DNA technology was developed more than 40 years ago, no one could have imagined the impact it would have on both society and the scientific community. In the field of genetic engineering, the most important tool developed was the plasmid vector. This technology has been continuously expanding and undergoing adaptations. Here, we provide a detailed view following the evolution of vectors built throughout the years destined to study microorganisms and their peculiarities, including those whose genomes can only be revealed through metagenomics. We remark how synthetic biology became a turning point in designing these genetic tools to create meaningful innovations. We have placed special focus on the tools for engineering bacteria and fungi (both yeast and filamentous fungi) and those available to construct metagenomic libraries. Based on this overview, future goals would include the development of modular vectors bearing standardized parts and orthogonally designed circuits, a task not fully addressed thus far. Finally, we present some challenges that should be overcome to enable the next generation of vector design and ways to address it.

## Introduction

**Figure 1:**
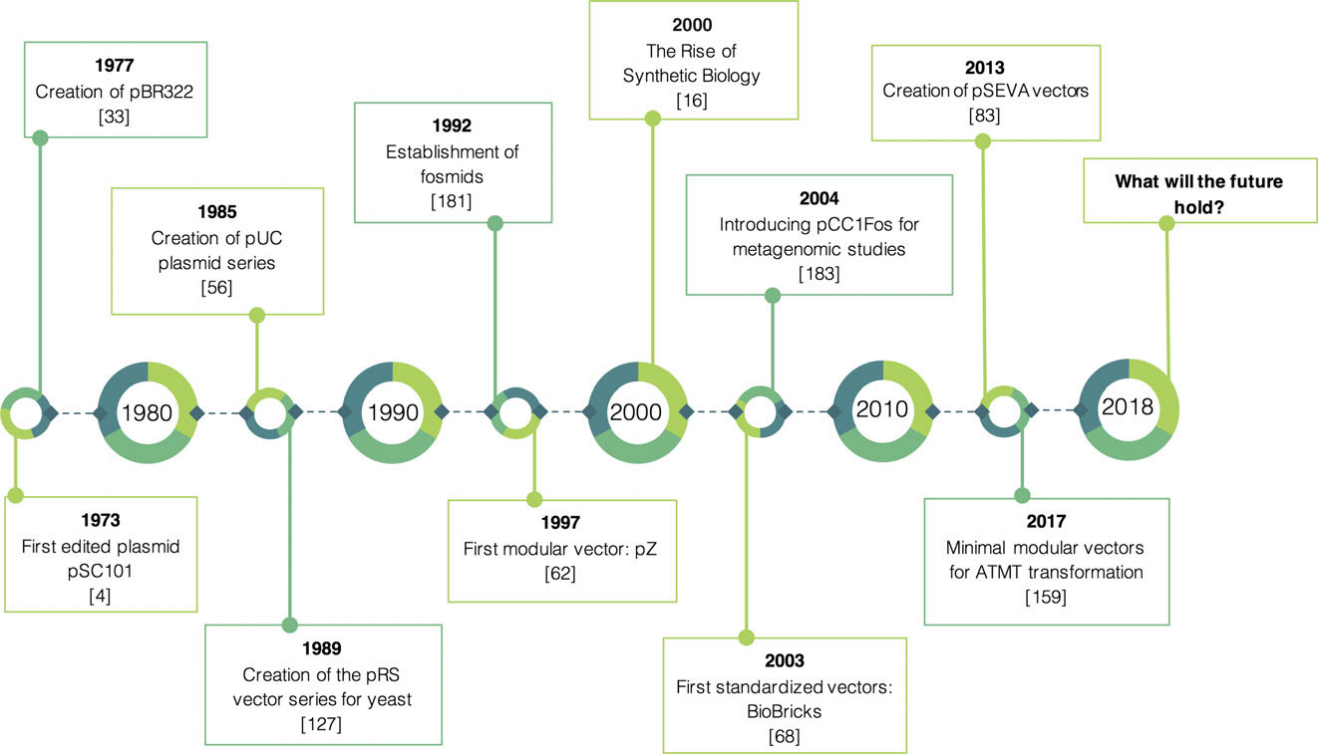
Timeline showing the most decisive breakthroughs regarding vector technology and design from 1970 until the present.

In the past decades, plasmid vectors have become a pivotal tool in the field of molecular biology. They have allowed several major discoveries and have become essential tools in both basic and applied science by providing novel elements for accessing the molecular features of life. Plasmids have also been used worldwide for bolstering biotechnological advances, from the production of insulin by a recombinant strain of *E. coli* to treat diabetes (Johnson, 1982) to corn crops containing a *Bacillus thuringiensis* gene (Koziel *et al*., 1993). Joshua Lederberg coined the term ‘plasmid’ in his work on cytoplasmic heredity published in 1952 (Lederberg, 1952). This term has been widely accepted and used with the understanding that these genetic elements are not organelles, individual genes, parasites (viruses) or symbionts. The first time a plasmid was edited was in 1973 when researchers exchanged a gene for tetracycline resistance from pSC101 to a kanamycin one, becoming pSC102 (Cohen *et al*., 1973). Later, pBR322 was constructed and used as the base module for the engineering of a number of different genetic tools, many of them summarized in this review. Hence, plasmids became vectors, as means of transportation, for delivering and manipulating foreign DNA inside a host cell, starting the new era for molecular biology. The first edition of ‘Molecular cloning: the laboratory manual’, the reference book in most molecular biology laboratories, was published in 1982 and it marks the use of vectors as probably the most important tools for genetic manipulation. Therefore, scientists can now understand the behaviour, physiology, molecular mechanisms and gene expression patterns of cells and organisms, all due to increasing development of new molecular cloning strategies. A timeline for remarkable discoveries in vector design and technologies is presented in Fig. 1.

**Figure 2:**
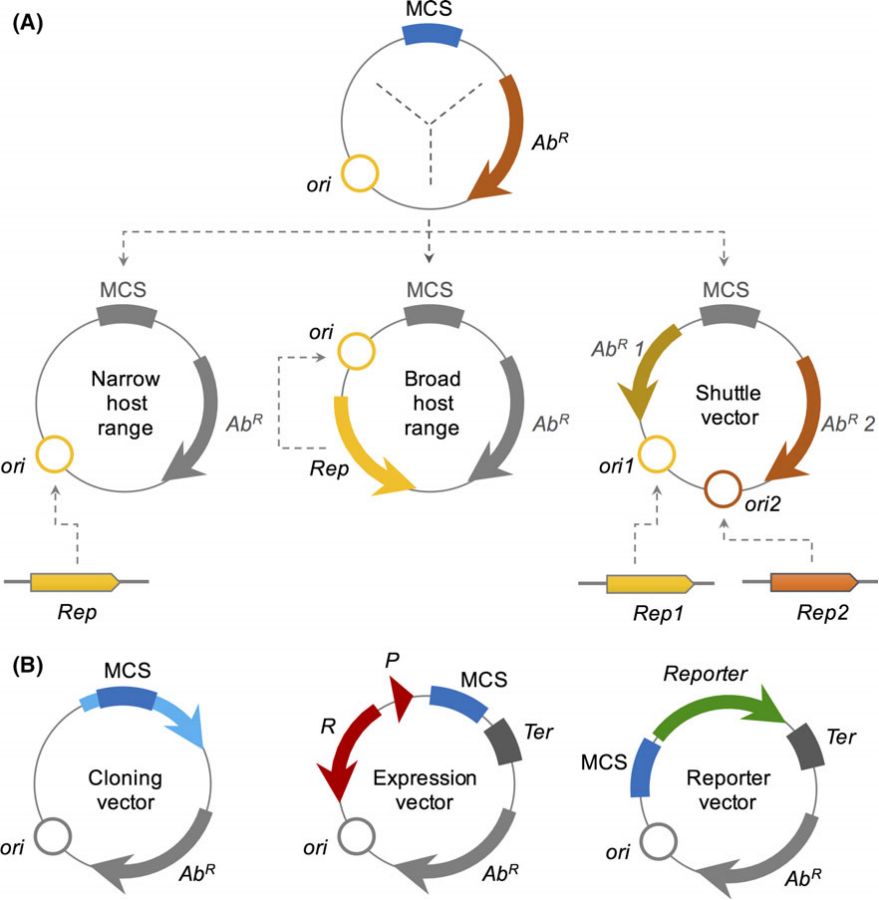
Most common bacterial plasmid architectures and categories.A. On the top, a minimal general architecture is formed by an origin of replication (*ori*), an antibiotic resistance marker (*Ab* ^*R*^) and a multiple cloning site (MCS). From this general architecture, vectors can be categorized as narrow‐host‐range, broad‐host‐range and shuttle vectors. In the case of narrow‐host‐range, the *ori* relays on the replication machinery or a protein (*Rep*) provided by the host. In the case of broad‐host‐range vectors, the plasmid harbours its own Rep gene, which makes it mostly host‐independent. In the case of shuttle vectors, two *ori* regions (*ori1* and *ori2*) are placed in the vector, each one being recognized by a specific *Rep* protein from different hosts (Rep1 and Rep2). In addition to that, two Ab^R^ (Ab^R^ 1 and Ab^R^ 2) are introduced to allow the selection in the appropriate host.B. Different functionalities of vectors. In cloning vectors, a MCS is usually located within a selection marker (such as the *lacZα* gene in pUC vectors) to allow the easy identification of plasmids with inserted fragments. In expression vectors, the MCS is preceded by an expression system (here shown as a regulated promoter P and its cognate regulator R) that allows the expression of the cloned fragment in response to a chemical inducer. Additionally, a strong terminator signal (*Ter*) is located after the MCS to ensure efficient transcriptional termination and increased plasmid stability. Finally, in a reporter vector, a reporter gene (such as a fluorescent protein, a luciferase coding gene or an enzyme‐coding gene such as *lacZ*) is flanked by an MCS and a strong terminator. In this system, the cloning of a promoter sequence in the MCS allows the investigation of promoter dynamics using the reporter gene of choice.

In general, vectors should have a set of characteristics that make them suitable for transformation and selection in the host organism. The first component is the origin of replication (*ori*) that will be recognized by the cellular replication machinery and will also define the number of copies of a given plasmid in the cell. Replication origins are usually recognized by their specific organism in what is called the narrow‐host‐range vectors, but there is also a category of broad‐host‐range vectors that contain origins capable of replicating in more than one species or genus, since they encode the protein that recognizes their own replication origin inside the plasmid (Durland *et al*., 1990). Furthermore, there is a series of vectors called ‘shuttle vectors’ that contain two different origins and two different selection markers so they can be transformed into two distinct organisms (Struhl *et al*., 1979). The differences between the ranges of hosts are assessed in Fig. 2A. It is important to notice that if the final plasmid has a proper origin of replication for the host, the genetic material inserted in the organisms is stable and can replicate autonomously. However, if no origin is available, the transformation requires the recombination of the vector into the host chromosome (e.g. in suicide plasmids), which necessarily leads to genome modification. Therefore, the existence of efficient origin of replications allows the decoupling between transformation and genome modification.

Another crucial component for a vector is the selection marker, which can be any gene allowing a selective advantage to the positive transformants, ranging from auxotrophy (corresponding to a metabolic enzyme missing in the host genome) to drug resistance (Gnügge and Rudolf, 2017). Also, a multiple cloning site (MCS) is usually added to facilitate cloning of the desired DNA, containing several sites recognized by different restriction enzymes. For simplicity, vectors can be divided into three typical classes most commonly used by researchers: cloning vectors, expression vectors and reporter vectors (Fig. 2B). Cloning vectors are the ones used to make numerous copies of a DNA of interest, keeping them stable inside a host organism (Rodriquez and Denhardt, 1988; Shizuya *et al*., 1992). Expression vectors are used to produce large amounts of a protein of interest; they usually contain a regulator and a target promoter that controls the expression of the gene encoding that protein (Stanley and Luzio, 1984; Terpe, 2006). Additionally, in reporter vectors, it is possible to place the promoter of the gene of interest to modulate a reporter protein, which can be fluorescent (Shaner *et al*., 2005), luminescent (Winson *et al*., 1998) or enzymatic, such as the ß‐galactosidase assay (Juers *et al*., 2012), among others. These reporter vectors allow *in vivo* analysis of gene expression kinetics throughout the organism's growth, allowing sophisticated studies even at the single‐cell level (Elowitz *et al*., 2002).

As new technologies and methodologies are surfacing, and researchers are now eager for fast, enhanced and easy‐to‐use molecular tools, mastering the principles and technologies of vector design has become a fundamental challenge. This is making room for the rise of an entirely novel discipline called synthetic biology (Rawis, 2000). This innovative field of study combines biological parts and modules to create more reliable and robust systems (Purnick and Weiss, 2009). The recent advances in DNA manipulation techniques such as automated DNA synthesis, sequencing and assembly have been combined with the synthetic biology framework, providing new perspectives on vector design and construction.

Not only did the majority of fundamental findings regarding molecular cloning arose from lessons given by microorganisms, but also there is an immense and much unexplored potential of those organisms in a wide range of applications such as biofuels and fine chemicals production (Sheldon, 2014; Jullesson *et al*., 2015; Kircher, 2015), biosensors (Courbet *et al*., 2015), bioremediation (Gavrilescu *et al*., 2015) and biomedical therapies (Din *et al*., 2016). Thus, efforts to understand and manipulate microorganisms are imperative, and as we acquire more knowledge, the classical genetic engineering approaches are no longer sufficient to answer all questions. Another challenge dwells in all the microorganisms that cannot be cultivated. Targeting that necessity, in 1998, Handelsman *et al*. launched a new approach called Metagenomics (Handelsman *et al*., 1998). The goal of metagenomics is to bypass the ‘pure culture paradigm’, which has blinded researchers from a genuine view of the microbial world for a long time. In fact, ‘meta’ is Greek for ‘transcendent’, meaning that pure genomics is not enough to understand all of Earth's diversity (Handelsman and Tiedje, 2007). To confront that task, several systems had to be adapted and created to clone DNA from environmental samples inside a cultivable host to search for genes of interest. This opens a new path towards the development of modern machinery for optimizing the prospection of novel biological parts through functional metagenomics (Guazzaroni *et al*., 2015; Alves *et al*., 2017a).

Despite the technological advances, the current vector architectures are still far from the conceptual framework. This is due to a number of caveats ranging from the intrinsic complexity of biological systems to a lack of solid standards for vector design. To address that, this review intends to condense most of the knowledge from the beginning of molecular cloning until the modern, advanced vectors used to transform microorganisms from bacteria to filamentous fungi, including their applications in metagenomics. In this sense, the present review aims to gather as much information regarding the available systems as possible, establishing a systematic overview of the uncontrolled expansion of genetic tools for microbiology accessible today, highlighting its current state along with the main challenges we still face. Thus, we provide an insight into how sophisticated current vectors are and glimpse into the new horizon of ever‐evolving tools we still need to generate.

## Development of vectors to engineer bacteria

Extrachromosomal genetic elements, now widely known as plasmids, were first recognized in bacteria over 60 years ago (Cohen, 2013; Kado, 2014). The development of recombinant DNA techniques in the 1970s (Lobban and Kaiser, 1973; Novick *et al*., 1976; Bolivar *et al*., 1977; Sinsheimer, 1977; Cohen *et al*., 1992; Cohen, 2013; Kado, 2014) led to a multitude of possibilities for manipulating those natural plasmids, turning them into vector systems which could be useful in several applications. Early vector systems were based on natural ColE1 derivatives and were primarily restricted to *E. coli* owing to their replication machinery (Hershfield *et al*., 1974; Bolivar *et al*., 1977). The introduction of broad‐host‐range plasmids such as RK2 (from *Pseudomonas aeruginosa*; Stalker *et al*., 1981; Thomas, 1981) and RSF1010 (from *Salmonella panama*; de Graaff *et al*., 1978; Bagdasarian *et al*., 1981) made it possible to introduce recombinant DNA technologies into bacteria other than *E. coli*. In recent times, a number of vectors have been derived and gradually optimized (for properties such as size, stability and functionality) from those naturally occurring plasmids for a myriad of purposes.

One of the first and most significant artificial vectors developed was the pBR322 (Bolivar *et al*., 1977) which was derived from *ColE1*. This vector, still currently in use, was initially built for general cloning purposes and can be considered one of the most important bacterial vectors, since several other tools have been derived from it for a wide range of functions (Balbás *et al*., 1986; Rodriquez and Denhardt, 1988). Some structural and functional modifications include the addition of new restriction sites (Davison *et al*., 1984; Sambrook *et al*., 1989), differentiation or change of selection marker (Rao and Rogers, 1979; Herrin *et al*., 1982; Richardson *et al*., 1982), increased stability (Skogman *et al*., 1983; Summers and Sherratt, 1984; Zurita *et al*., 1984; Chiang and Bremer, 1988), change in the copy number (Twigg and Sherratt, 1980; Boros *et al*., 1984; Soberon *et al*., 1990), addition of a signal peptide to facilitate protein secretion (Villa‐Komaroff *et al*., 1978; Talmadge and Gilberg, 1980), and finally, change in the origin for one of a shuttle vector, allowing vector propagation in different hosts (Brückner, 1992). Nowadays, two important and widely used commercial cloning vectors are pJET1.2/blunt (Thermo Fisher Scientific) and pGEM‐T Easy (Promega Corporation), with features such as lethal phenotype and β‐galactosidase activity‐driven selection respectively. As these vectors can supply most requirements for simple cloning processes, few efforts have been made over the past years in the attempt to create novel tools for this task.

On the other hand, gradual development and optimization of expression vectors have potentially allowed the biosynthesis of any type of heterologous proteins *in vivo*. In this context, the pUC‐plasmid series for expression (Norrander *et al*., 1983; Hanna *et al*., 1984; Yanisch‐Perron *et al*., 1985), one of the pBR322 derivatives, marked the timeline of expression vectors. The pUC‐series vectors are mainly composed of a *lac* promoter–operator and require compatible hosts for α‐complementation (blue/white screening system that allows recovering of functional β‐galactosidase LacZ), providing a positive selection for recombinants. In the same way cloning vectors were successively derived from each other, many expression vectors were derived from pUC‐series as the lacUV5 mutant (Rodriquez and Denhardt, 1988), which contains just two base pair mutations in the −10 hexamer of the classical *lac* promoter and the *tac* hybrid promoter (Rodriquez and Denhardt, 1988). Many promoter/operator modifications were made during this time for addressing specific needs in protein expression systems. In addition to pUC‐based vectors, a number of other expression vectors were constructed from the *trp*‐promoter, carrying different segments of the *trp* operon (Enger‐Valk *et al*., 1980; Hallewell and Emtage, 1980). Nowadays, besides pUC18 and pUC19, the pET‐series (Novagen, Madison, WI, USA) vectors, which were also derived from pBR322 (Ramos *et al*., 2004) and pGEX (GE Healthcare Life Sciences), are widely used because they are high copy number expression vectors that contain protein tags that facilitate the subsequent purification of the desired protein. These two vectors are best suited for quick and easy heterologous protein expression.

Although the diversity of bacterial vectors has enormously increased during the decades following the discovery of the recombinant DNA technology, the design and architecture of those tools occurred rather unsystematically. Vectors with similar functions were generated with different standards between laboratories, hindering data comparison and requiring fastidious efforts for re‐cloning sequences into available vectors. In addition, although a few plasmids were developed for different hosts, most of them were focused on the organism model *E. coli*, which is not ideal for many biotechnological applications (e.g. fine chemical production, biodegradation and environmental release). Furthermore, the lack of standards for minimal genetic units such as promoters, terminators and replication origins (usually based on the amplification of naturally occurring sequences) generated vectors with long and unnecessary sequences, sometimes bearing undesired features such as common restriction sites and cryptic functions, which could interfere with the plasmid integrity/stability (Summers and Sherratt, 1984). Thus, despite the massive amount of information accumulated regarding bacterial vectors, the field has drifted towards structural diversification rather than to a more unified standard.

### The era of modular vectors in bacteria

**Figure 3:**
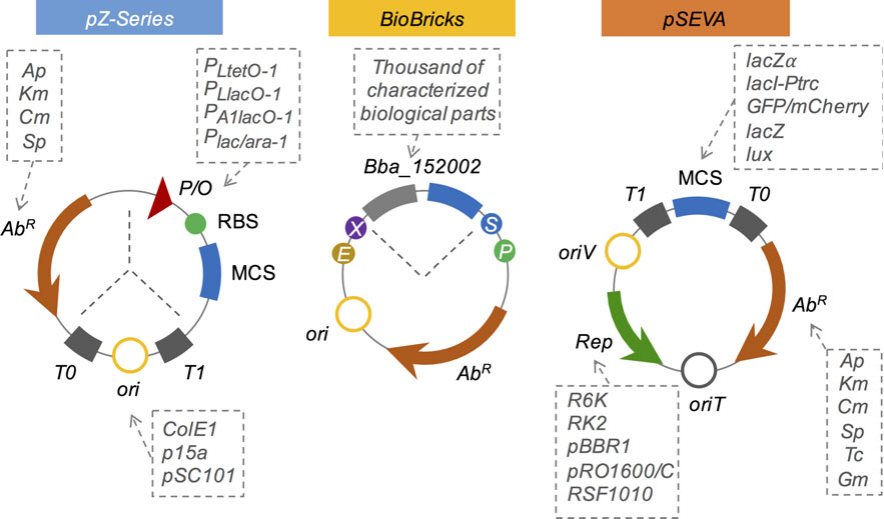
New generation of modular vectors. The pZ‐series represented remarkable progress in the new generation of vectors. In this set‐up, three modules are combined to generate a collection of vectors. In the original design, three narrow‐host‐range origins were combined with four antibiotic resistance markers and a few expression systems and reporters (Lutz and Bujard, 1997). In the BioBrick platform, a very interactive set‐up is used to assemble complex circuits by the joint of prefix and suffix fragments using only four restriction enzymes (*Eco* RI,* Xba*I, *Spe*I and *Pst*I). This platform is merged with a collection of thousands of well‐characterized biological parts and is widely used by the iGEM community (Knight, 2003; Shetty *et al*., 2008). In the SEVA platform, broad‐host‐range origins of replication (represented by the *oriV* and Rep elements) are selected, allowing the replication of the plasmids in multiple Gram‐negative bacteria. This is combined with a number of antibiotic resistance markers and many functional systems at the MCS regions (some examples are shown in the figure). Another exclusive feature of this platform is the existence of a transference origin (*oriT*) to allow the mobilization of the plasmids to the target bacteria using conjugation (Silva‐Rocha *et al*., 2013).

In the recent years, further advances in molecular cloning process, computational methods and DNA sequencing automation have gradually allowed the scientific community to overcome the challenges of vector design. A cornerstone in generating a standardized system was the creation of pZ vectors in 1997 for the study of transcriptional regulatory elements in bacteria (Lutz and Bujard, 1997). In this work, Lutz and Bujard (1997) introduced the term ‘modularity’ in vector design. Modularity has been widely used in studies of technological and organizational systems. Product systems are deemed ‘modular’, for example, when they can be decomposed into a number of components that may be mixed and matched in a variety of configurations (Baldwin and Clark, 2000). In this sense, the pZ vectors were organized into three main functional modules, physically separated by specific restriction sites: (i) antibiotic resistance module, (ii) origin of replication module and (iii) expression/transcriptional regulated module. Each part of the system could be easily shifted by a functional variant and combined to the next one, allowing the generation of a wide range of testable vectors. The result was a library of unique vectors composed of different components, yet bearing the same architecture. The central point of this system was to evaluate the efficiency of previously described transcriptional elements (Knaus and Bujard, 1990; Skerra, 1994; Guzman *et al*., 1995) in regard to plasmid features such as copy number and antibiotic resistance. The use of the promoters controlled by elements of the *lac*,* ara* or *tet* operon (Tn10) and therefore induced by IPTG, arabinose and tetracycline, respectively, generated strongly repressible promoters that could regulate expression levels up to 5000‐fold. In addition, using different origins of replication was shown to shift expression tightness (Lutz and Bujard, 1997). Therefore, those findings represented a major advance not just in the study of gene regulation, but also in the study of how the components or functional modules of a vector system affect each other (Fig. 3).

A few years after Lutz and Bujard's work, despite the rather tumultuous generation of cloning vectors, a new conceptual framework was conceived, combining the technological advances in molecular biology and the hierarchical abstraction and rational design concepts from Engineering Sciences. It was called synthetic biology (Andrianantoandro *et al*., 2006) and its main rationale was to shift molecular biology from traditional ‘copy and paste’ methods to a more high‐throughput, concise and design‐oriented background for generating novel biological functions. As highlighted by Lutz and Bujard's studies (Lutz and Bujard, 1997) and by many others afterwards, each part of the vector system matters, and in order to standardize biological parts, the generation of vectors with easily interchangeable modules would be essential. Thus, it did not take long for the first library of standardized vectors and biological parts to be established. In 2003, the BioBrick standard (BBF RFC 10) was proposed by Knight (2003), employing standard suffix and prefix sequences that flanked every designed biological part (see Fig. 3). This method was further optimized (Canton *et al*., 2008; Shetty *et al*., 2008) and has allowed an easy process for joining parts by standard restriction‐ligation methods and hierarchical assembly (Shetty *et al*., 2011). Further efforts driven by the dissemination of the community‐based iGEM competition have allowed the development of a repository of standard biological parts and novel standards for vector assembly (Endy, 2005; Knight, 2007; Müller *et al*., 2009; Røkke *et al*., 2014).

Although the BioBricks standard was essential for establishing functional units in synthetic biology such as promoters, terminators and genes, it did not provide many flexible modules (e.g. no easily and independent interchangeable modules for origins of replication and antibiotic resistance genes). Furthermore, the traditional restriction enzyme‐based method required the previous removal of standard recognition sites from the biological part and generated an 8‐bp scar during the assembly process, which could destabilize the plasmidial system (Anderson *et al*., 2010; Ellis *et al*., 2011; Yao *et al*., 2013). In order to overcome these issues, a few other systems with the same basic architecture were designed, such as the Bgl Bricks (Anderson *et al*., 2010; Lee *et al*., 2011), the iBricks (Liu *et al*., 2014) and the epathBricks (Xu and Koffas, 2013), the latter being the most modular system among them. The pBAM1 vectors (Martínez‐García *et al*., 2011), a modular remake of the original mini‐Tn5 transposon vector concept, and its more versatile and successful successor, the Standard European Vector Architecture (SEVA; Silva‐Rocha *et al*., 2013), also use a set of restriction sites to standardize DNA assembly (see Fig. 3). The SEVA collection differs from the majority of assembly methods in that it is more correctly described as a modular standard. This describes a set of criteria for the physical assembly of plasmids according to a three‐component architecture: an origin of replication segment, a selection marker segment and a cargo segment (Silva‐Rocha *et al*., 2013). These segments are flanked by insulator sequences and are assembled together with a set of rare restriction sites (Fig. 3). Although some optimizations were made over the years, such as the SEVA linkers (Kim *et al*., 2016), the SEVAs still suffer from a few issues such as rare restriction enzyme sites, non‐optimized synthetic parts and a lack of standardization for some of its functional parts.

While the rationales behind traditional restriction site‐based assembly methods support modularity, their limitations have led several research groups in the synthetic biology community to ‘trade‐in’ standardization and modularity for ‘bespoke’ assembly methods that enable one‐pot assembly of multiple DNA parts. Those methods for *à la carte* vector assembly have become increasingly popular along the last decade due to their versatility, low cost and high speed for simultaneous assemblies. Although very diversified, the most common methods are Gateway (Invitrogen, 2011), Golden Gate (Engler *et al*., 2009), MoClo (Weber *et al*., 2011), Gibson (Gibson *et al*., 2009), Slic (Li and Elledge, 2007), Cpec (Quan and Tian, 2009), Slice (Zhang *et al*., 2012) and Paperclip (Trubitsyna *et al*., 2014). For a more detailed review, see (Kelwick *et al*., 2014; Casini *et al*., 2015). However, it is important to highlight that although vector modularity has gradually shifted towards novel high‐throughput assembly methods, well‐defined modular vectors are still essential tools for many applications. Here, we can emphasize the use of modular vectors for the exploration of genetic functional space in bacteria (Westmann *et al*., 2018), the functional re‐wiring of genetic features and the generation of novel biological functions. The concept of modular vectors has also inspired the development of plasmid collections for specific bacterial hosts such as *Pseudomonas putida*,* Clostridium* spp., *Cyanobacteria* spp., *Bacillus* spp. and *Geobacillus* spp. (Heap *et al*., 2009; Radeck *et al*., 2013, 2017; Silva‐Rocha *et al*., 2013; Taton *et al*., 2014; Wright *et al*., 2015; Reeve *et al*., 2016; Popp *et al*., 2017), which become essential for establishing novel chassis in synthetic biology.

Thus, we have reached a state in which modular vector or high‐throughput assembled constructs, from vectors to genomes, can be easily designed and assembled through computational and experimental tools (Casini *et al*., 2015; Woodruff *et al*., 2017). However, it is clear that this powerful design and build process was not followed by the standardization of biological parts that ultimately compose those systems (Decoene *et al*., 2017). The lack of biological information regarding the behaviour of simple genetic features such as promoters, terminators and origins of replication hinders the potential of engineering living organisms. Furthermore, we are currently experiencing the age of emergent phenomena in synthetic biology (Pósfai *et al*., 2006; Kwok, 2010; Chen *et al*., 2015; Monteiro *et al*., 2017), an ubiquitous property of life, highlighted decades ago by systems biology (Bhalla and Iyengar, 1999; Kitano, 2002). In this context, the most well‐characterized biological part might exhibit unpredictable behaviour when combined with other parts or when exposed to different compositional contexts such as the constraints imposed by vector biophysical structure/architecture or the combination of functional regulatory *cis*‐elements (Cardinale and Arkin, 2012; Goñi‐Moreno *et al*., 2017; Maria Oliveira Monteiro *et al*., 2017; Yeung *et al*., 2017). Therefore, the new frontier in vector design is to understand the constraints imposed by its minimal parts on a systemic context, allowing the fine‐tuning of its functionalities.

Moreover, an important step is to ensure that the massive amounts of data generated on biological parts and devices do not end up disconnected, and for that, standardization in data sharing is needed. To this end, various data registries and repositories of parts and devices have already been established and are curated regularly. Noteworthy examples are the Virtual Parts Repository (URL: http://sbol.ncl.ac.uk:8081/; Cooling *et al*., 2010), the Registry of Standard Biological Parts (URL: http://parts.igem.org/; Endy, 2005), the Joint BioEnergy Institute's Inventory of Composable Elements (JBEI‐ICE; URL: https://acs-registry.jbei.org; Huynh and Tagkopoulos, 2016), the Standard European Vector Architecture 2.0 database (SEVA‐DB 2. 0, URL: http://seva.cnb.csic.es/; Martínez‐Garćía *et al*., 2015), and newly the Plant Associated and Environmental Microbes Database (PAMDB; URL: http://genome.ppws.vt.edu/cgi-bin/MLST/home.pl). Some repositories, such as the Registry of Standard Biological Parts, have also implemented quality control checks.

The crude, yet essential, generation of bacterial vectors back in the early days has been continuously refined over the years, following the development of novel technologies and molecular techniques. The subsequent advent of synthetic biology and novel assembly methods has driven the field of bacterial vector design to a whole new level. The previously proposed concept of modularity was finally incorporated into biological engineering, allowing a number of standard collections to be derived from it. Currently, vector modularity, although essential, is at stake with the novel high‐throughput technologies for *à la carte* DNA assembly (see Fig. 3). Still, directed approaches such as the rigorous standardization of biological parts, the comprehension of emergent properties on genetic circuits and the expansion of biological functions to a wide range of chassis will constitute the next frontier for advancing bacterial vector design.

## Evolution of vector engineering for fungi

### Tools for yeast transformation

Circular DNA was already known to be present in prokaryotes, as already mentioned, over 60 years ago. In eukaryotes, more precisely in *Saccharomyces cerevisiae*, circular DNA was first discovered in mitochondria, and it took several years for researchers to realize this was too prokaryotic DNA (Margulis and Chapman, 1998). A non‐mitochondrial natural plasmid DNA was first described in 1971 (Guerineau *et al*., 1971). Three years later, researchers found an antibiotic resistance gene present in those plasmids and realized they could behave just like the bacterial ones allowing DNA cloning (Guerineau *et al*., 1974). Soon after, yeast transformation was described as well as integration of the genes in the yeast genome (Hinnen *et al*., 1978; Cameron *et al*., 1983). Guerineau's group noticed that yeast plasmids had a length of about 2 μm, consequently, they were named 2μ. In the years that followed, many researchers began constructing vectors for yeast manipulation, many of which used the 2μ origin (Struhl *et al*., 1979; Ferguson *et al*., 1981).

**Figure 4:**
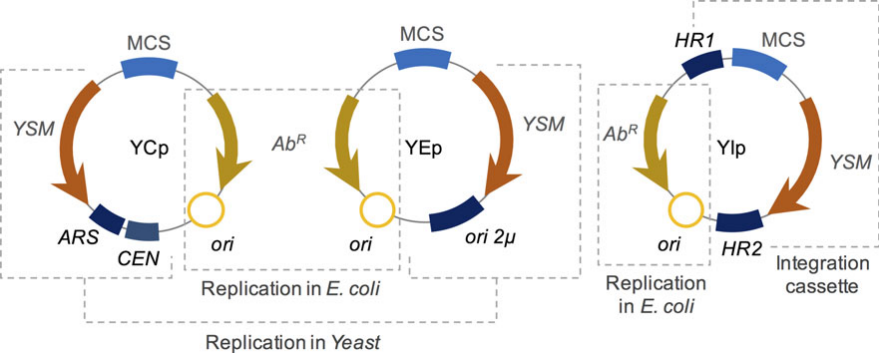
Modular vectors designed for yeast. The yeast centromeric plasmids (YCps) harbour the autonomously replicating sequences (ARS) and centromeric sequences (CEN), which allows the vectors to behave as mini‐chromosomes. The Yeast Episomal plasmids (YEps) are endowed with the 2μ origin of replication and are similar to plasmids in bacteria. In the case of Yeast Integrative plasmids (Yips), homologous regions (labelled as HR1 and HR2) to the host chromosome allow the integration of the target region through homologous recombination events. In all vectors, the yeast selection marker (YSM) represents a gene that allows the selection of transformants harbouring the vectors. In all cases, specific regions for replication of the bacterial host (usually *E. coli*) and the region required for replication or integration in yeast are highlighted.

Plasmids used to transform *Saccharomyces* can be divided into three groups: yeast centromeric plasmids (YCps), Yeast Episomal plasmids (YEps) and Yeast Integrative plasmids (Yips). The YCps need autonomously replicating sequences (ARS) and centromeric sequences (CEN) where kinetochore complexes attach, thus behaving like a microchromosome (Clarke and Carbon, 1980; Westermann *et al*., 2007). The YEps are based on the endogenous 2μ plasmid mentioned above with addition of a bacterial origin of replication and selection marker, yeast selection marker and the expression cassette. Still, the YIps need to have homology sequences so they can integrate in the yeast genome via homologous recombination (Gnügge and Rudolf, 2017). For all of them, the selection marker is usually auxotrophic, which somewhat restricts their use because there is usually only URA3 (encoding orotidine‐5′‐phosphate decarboxylase), LEU2 (encoding 3‐isopropylmalate dehydrogenase), HIS3 (encoding imidazoleglycerol‐phosphate dehydratase) and TRP1 (encoding phosphoribosylanthranilate isomerase) options, besides the need for a strain with the original gene deleted. Generally, all series of vectors present the three types; in a matter of deciding which methodology to use, the biological question being examined should be considered (Fig. 4).

Despite the fact that it was Hinnen and colleagues that developed a transformation method for yeast using bacterial vectors in 1978, the vectors containing the ability to replicate in both *E. coli* and *S. cerevisiae* were developed a year later by Struhl *et al*. (1979). Shuttle vectors, as they are called now, are still the preferred method for yeast transformation, and they are constantly evolving. Ferguson *et al*. (1981) developed the series of pRC1, pRC2 and pRC3 derived from pKC7 (which was originally derived from pBR322). Gietz and Sugino (1988) built the plasmids YCplac, YEplac and YIplac based on pUC19 in 1988. Around the same time, Ma *et al*. (1987) developed a method of easy recombination of plasmid parts and established the series of shuttle vectors YCp400 and YEp400. All of these vectors were considerably large (some more than 10 kb) and had no more than 10 unique restriction sites for cloning. However, in 1989, there was the breakthrough for yeast scientists: the pRS series (Sikorski and Hieter, 1989). These vectors were made small (around 5 kb), with several restriction sites (around 13 unique sites each), four different selection markers to choose from, and since they were based on pBLUESCRIPT, positive transformants could be selected by colour in *E. coli*. pRS vectors were such a turning point in this field that new designs for yeast tools were very limited for almost twenty years afterwards, with scientists only adding some adaptations such as new resistance markers (Chee and Haase, 2012), and these tools are still frequently used and adapted (Avalos *et al*., 2013).

Nevertheless, in the last 10 years, huge improvements have been made in yeast molecular tools. The plasmids are now smaller and have the feature of recycling selection markers to overcome the lack of variability of those. In 2007, the pAG series was created, still based on the pRS series, containing more than 200 options of YCps, YEps and Yips vectors for cloning, expression and also presenting reporter genes such as GFP and dsRed (Alberti *et al*., 2007). In 2011, Fang *et al*. (2011) developed a collection called pXP based on pUC18, with the advantage of a selection marker flanked by *loxP* sites, meaning it can be recycled using *loxP*/Cre technology (Güldener *et al*., 1996). Three years later, in 2014, Jensen and his group designed the EasyClone: a set of vectors that can integrate three cassettes at a time carrying up to two genes each and showed that this is more homogeneous and stable than expressing more than one gene in episomal vectors (Jensen *et al*., 2014). A variation of EasyClone called EasyClone2.0, published in 2015, has the very unique feature of using dominant markers for selection instead of auxotrophy, so it can be applied to prototrophic strains (Stovicek *et al*., 2015). A year later, the EasyCloneMulti collection was created, complementing the latter two. EasyCloneMulti integrates into long terminal repeats (LTR) of Ty retrotransposon sequences, enabling multiple integrations throughout the yeast genome (Maury *et al*., 2016). All of these three series of EasyClone vectors contain USER cloning sites (Bitinaite *et al*., 2007) as a facilitator strategy and present the benefit of *loxP*/Cre recycling systems as well.

At last, the pRG series was generated very recently by Gnügge *et al*. (2016). This collection comprises vectors of all types – YEps, YCps and Yips – exhibiting the unlimited benefit of modularity, which means that all parts of the vectors are flanked by restriction sites and can be interchanged or substituted, as mentioned earlier. The systems also allow multiple integrations and their resistance markers are auxotrophic. The integration vectors of both EasyClone and pRG series use a double cross‐over mechanism to integrate into the genome, thus determining the stability of the insert inside the chromosome. Table 1 summarizes the most important tools for yeast manipulation from 2007 to 2017. Likewise, Gnügge and Rudolf (2017) also described, in an extended review, most of the shuttle vectors available for yeast.

**Table 1:** Plasmid vectors used for fungal transformation

Plasmid series name	Fungal selection marker	Approximate average vector size	Features	Fungi type	References
pAG	HIS3, LEU2, TRP1, URA3	7 kb	More than 200 options; contains fluorescence reporters	Yeast	Alberti *et al*. (2007)
pXP	HIS3, LEU2, MET15, TRP1, URA3	5 kb	Recycling of selection markers by loxP/Cre technology	Yeast	Fang *et al*. (2011)
EasyClone	HIS3, LEU2, LYS5, URA3	6 kb	Multiple integrations; recycling of markers.	Yeast	Jensen *et al*. (2014)
EasyClone2.0	*amds, ble, dsd, hph, kan, nat*	6 kb	Compatible with prototrophic strains; recycling of markers	Yeast	Stovicek *et al*. (2015)
EasyCloneMulti	*Kl*.URA3‐*degradation signal*	6 kb	Integrates into Ty sequences; recycling of markers.	Yeast	Maury *et al*. (2016)
pRG	HIS3, LEU2, LYS2, MET15, URA3	6 kb	Modular design; multiple integrations; recycling of markers	Yeast	Gnügge *et al*. (2016)
pWEF	*hph*	12 kb	Binary vector	Filamentous	Lv *et al*. (2012)
pDESTR	*hph*	5 kb	Gene targeting and disruption	Filamentous	Abe *et al*. (2006)
pCBGW‐GFP	*hph*	8 kb	Expression vector	Filamentous	Zhu *et al*. (2009)
pGWB2‐GFP	*hph*	Not shown	Binary vector	Filamentous	Zhu *et al*. (2009)
pEX1 and pEX2	*pyrG*	10 kb	Binary vector	Filamentous	Nguyen *et al*. (2016)
pBI‐hph	*hph*	15 kb	Binary vector	Filamentous	Zhong *et al*. (2007)
pALS‐1	*qa‐2+*	13 kb	Tested in *N. crassa*.	Filamentous	Sthol and Lambowitz (1983)

In contrast with the classical vectors and taking advantage of the CRISPR/Cas feature of not needing a selection marker, Mans *et al*. (2015), Generoso *et al*. (2016), Shi *et al*. (2016) and Apel *et al*. (2017) developed tools for efficiently editing the *S. cerevisiae* genome. Similarly, the EasyClone series already has a new version of CRISPR/Cas vectors named EasyClone‐MarkerFree (Jessop‐Fabre *et al*., 2016). These are whole new strategies that can also be availed by researchers to study the characteristics of yeast cells.

*Saccharomyces cerevisiae* is a model organism with a very well‐annotated genome, thus it has been essential for the evolution of the ever‐growing field of genetic engineering (Nielsen *et al*., 2013). However, genetic tools such as vectors and integrating cassettes have been developed for non‐conventional yeast as well, considering their rising importance in biotechnology, such as *Pichia pastoris* (Cereghino and Cregg, 2000), *Kluveromyces lactis* (Van Ooyen *et al*., 2006) and *Yarrowia lipolytica* (Bredeweg *et al*., 2017). For an expanded review of non‐conventional yeasts, tools refer to Wagner and Alper (2016). Undoubtedly, efforts to construct yeast tools have largely focused on *Saccharomyces* for decades. However, the need for alternative and more adapted species of yeasts, for example *Kluyveromyces marxianus* that is thermo‐tolerant and can be used in bioreactors (Nambu‐Nishida *et al*., 2017), is accelerating the search for adaptable genetic systems. Considering this, we are probably only a few years away from achieving a robust yeast vector. These vectors are becoming smaller, easy to manipulate, modular and capable of multiple integrations. Additionally, they have options for recycling selection markers and most of them currently contain several restriction sites as cloning options. Still, the ideal framework would be a standard vector that could work in several (if not all) yeast species.

### Tools for filamentous fungi

Since genetically manipulating filamentous fungi is considerably more complex than manipulating yeast, much discussion has been raised to improve the molecular tools for genetic transformation of filamentous species. In this sense, some efforts have been made in this field to unravel the genetic mechanisms for successful cell transformation using fungal plasmids. A remarkable study in *Neurospora crassa*, a model fungus for genetic research, has helped determine how plasmids could be extended to successful fungal manipulation. The shuttle vector pALS‐1, originally described by Sthol and Lambowitz (1983), is a 13.1 kb recombinant plasmid that replicates in both *N. crassa* and *E. coli;* it is based on the backbone of the mitochondrial plasmid P405‐Labelle and on the *E. coli* plasmid pBR325, and also contains the *Neurospora qa‐2+* selection gene (Sthol and Lambowitz, 1983). This vector was one of the landmark genetic tools for fungi manipulation because it was described as one of the first vectors shown to replicate autonomously in the nucleus or in the cytosol of a filamentous fungus cell, which highlighted the field of fungal genetics and spread opportunities for creating versatile tools for this purpose (Sthol and Lambowitz, 1983).

**Figure 5:**
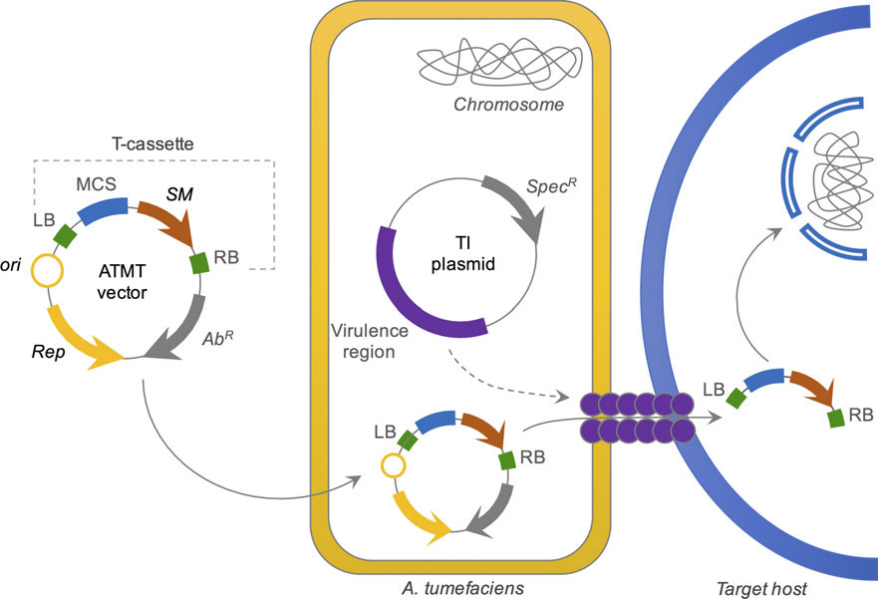
Minimal genetic tools based on *Agrobacterium tumefacien*‐mediated transformation (ATMT). ATMT vectors are based on broad‐host‐range plasmids and harbour a T‐cassette, which is composed of a MCS and a selection marker (SM) flanked by the left and right borders (LB and RB) required for the recognition of the *A. tumefaciens* machinery. Once inserted in the proper *A. tumefaciens* strain harbouring the TI plasmid (which expresses the components for T‐cassette mobilization), this vector can be used to introduce the T‐cassette into hosts such as fungi and plants.

Years later, in *Trichoderma reesei,* the most utilized fungus for cellulase production, Steiger *et al*. (2011) obtained a successful genetic transformation system that favoured homologous recombination using a *loxP*/Cre system for creating gene deletion with pMS plasmids vectors (Steiger *et al*., 2011). In this same fungus, Lv *et al*. (2012) reported the construction of two expression vectors, pWEF31 and pWEF32, with the cellobiohydrolase gene I (*cbh*I) promoter regulating the expression of a reporter fluorescent red protein (Lv *et al*., 2012) and demonstrating an effective *Agrobacterium tumefacien*‐mediated transformation (ATMT) process (Fig. 5). Despite the promising outcomes observed in *T. reesei,* further studies are required to more deeply elucidate its genetic mechanisms for transformation effectiveness, showing that creating new plasmid vectors is pivotal for functional genomic studies in this very relevant species.

Some fungal plasmids have been created towards multifunctional roles in fungal transformation, from increasing of gene copy number to integrative recombination. In this sense, a series of plasmids have been constructed based on pDONR vectors (Gateway) through Gateway cloning technology using λ phage integrase proteins and attachment regions for recombination (Schorbele *et al*., 2013). These vectors were designed to attend different conditions and applications in fungal species, and for that reason, it allows selection by nutritional and drug resistance markers, showing to be a promising and a versatile genetic tool for genetically manipulating distinct fungi (Schorbele *et al*., 2013).

Gateway technology has been also utilized to create several other vectors. For example, the vector pTROYA allowed the utilization of a fungal vector for RNA interference approaches to construct the PAC1 mutant strain in *Colletotrichum gloeosporioides* and it was a potential tool for screens in non‐sequenced organisms (Shafran *et al*., 2008). Additionally, Gateway approaches were also used for developing the pDESTR vector, a plasmid based on the backbone of pGEM‐T easy (Promega) without its MCS region plus a Gateway cassette and a hygromycin resistance gene sequence, created to accomplish gene targeting and disruption in filamentous species (Abe *et al*., 2006). Zhu *et al*. (2009) used Gateway for the construction of expression vectors for ATMT (Zhu *et al*., 2009). Studies like this one that perform fungal cell transformation through ATMT are getting more visibility in the scientific community due to the higher transformant frequency when compared to usual methods like protoplast fusion. Other binary plasmids have been reported for ATMT processes, such as the vectors pEX1 and pEX2, with auxotrophic selection for *pyrG* from *Aspergillus oryzae* (Nguyen *et al*., 2016), and pBI‐hph, with selection for hygromycin (Zhong *et al*., 2007).

New advances have continuously been reported concerning vectors used for *Agrobacterium* transformation. Recently, the creation of a series of vectors was described for fungal transformation through ATMT based on the plant binary vector pCAMBIA2200, named by Nishikawa *et al*. (2016) as pFungiway. These vectors were created for two distinct purposes: to guarantee the expression of a gene under the regulation of a constitutive promoter and to promote the negative regulation of target gene expression through RNAi (Nishikawa *et al*., 2016). More recently, a set of synthetic, minimal and modular binary vectors for multiple transfer of T‐DNA was published in 2017 (Pasin *et al*., 2017). Despite being applied in plants, this is a frontier technology that can be expanded and adapted for fungal transformation in the future. Regardless, Table 1 summarizes some of the most important vectors built for fungal transformation.

CRISPR‐Cas9‐based vectors are also feasible in fungal genetic manipulation. In this concern, Nødvig *et al*. (2015) created four new vectors derived from the pFC330 vector with distinct fungal selectable markers (*pyrG, arbB,* ble^R^ or hyg^R^; Nødvig *et al*., 2015). At this point, authors successfully demonstrated the genetic transformation and genome engineering of *Aspergillus* species, suggesting that CRISPR‐Cas9 tools are efficient but still could be enhanced.

Filamentous fungi are by far the toughest organisms to transform and manipulate among all microbes (Ruiz‐Díez, 2002). Even with the advances towards the state‐of‐the‐art vector creation, fungal plasmids used for genetic engineering are still poorly understood, unreliable and inefficient, especially when compared to bacterial ones. Still, the increasing development of synthetic biology studies focused on fungi has strongly contributed to the rising and constant necessity of creating new tools for fungal applications in several areas of molecular biology (Amores *et al*., 2016). New forms of vector design, plus characterization and standardization of their parts, are crucially needed to promote a better understanding of fungal molecular mechanisms.

## Engineering new vector for Metagenomics

The term ‘metagenome’ was introduced in 1998 by Handelsman *et al*. (1998), and since its first use, this methodology became a powerful tool for analysing composite genomes of microbial communities and their potential products for novel biotechnological and pharmaceutical applications. In addition, the use of functional metagenomics has been shown to be effective for identifying new enzymes, antibiotics and other molecules derived from a variety of environments without the need for isolating and cultivating microorganisms in the laboratory (Courtois *et al*., 2003; Ferrer *et al*., 2012; Pozo *et al*., 2012; Thompson *et al*., 2013; Yang *et al*., 2016).

Despite the great potential of metagenomic approaches, some barriers have limited the discovery of new genes. The probability of identifying a particular gene depends on multiple factors that are intrinsically linked: the host‐vector system, the size of the gene, the recovered metagenomic DNA abundance, the screening method and the efficiency of heterologous expression of the gene in a substitute host (the most frequently used is *E. coli*; Gabor *et al*., 2004; Villegas and Kropinski, 2008; Uchiyama and Miyazaki, 2009). According to Gabor *et al*. (2004), only 40% of enzymatic activities can be recovered from random cloning in *E. coli*. This might be due to a number of factors that exist in heterologous genes that differ from those used by *E. coli*, preventing the host cell expression machinery from recognizing these signals, such as differences in translation initiation codons; in *E. coli*, the preferred translation initiation codon is AUG, whereas in some organisms, GUG and UUG are preferred (Villegas and Kropinski, 2008). In addition, differences in codon usage, promoters for transcription and ribosomal binding sites may lead to no detectable expression of the target genes. Furthermore, incorrect protein folding, toxicity of the gene product and an inability to secrete the gene product may be obstacles to identifying new genes in functional screenings (Ekkers *et al*., 2012). Choosing an appropriate vector for constructing a metagenomic library depends on various factors, such as the DNA size of the expected target compound of interest, whether a small‐ or large‐insert library is considered, the hosts that will be used in the screenings, as well as the screening tests (Ekkers *et al*., 2012).

**Figure 6:**
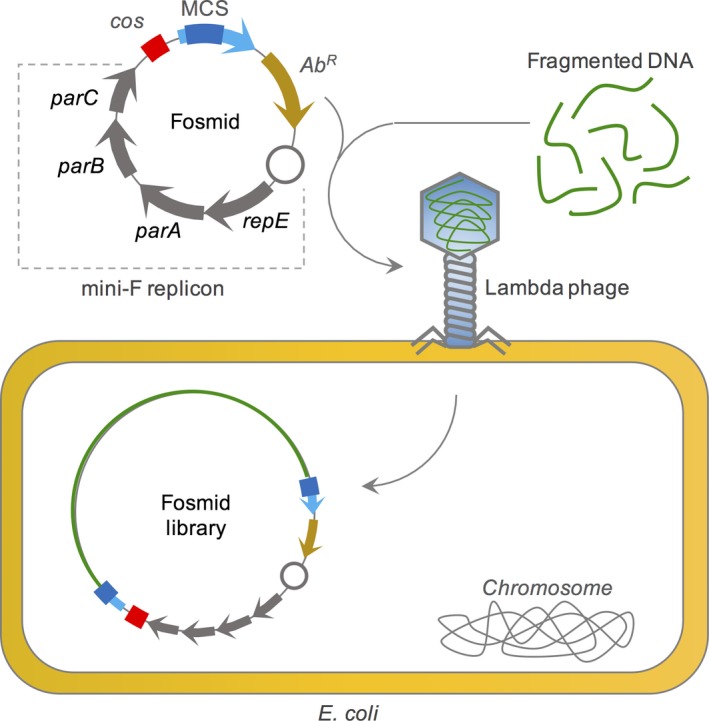
Tools based on minimal fosmids for cloning large DNA fragments. Fosmids are plasmid vectors based on the mini‐F origin of replication that harbour, in addition to MCS and Ab^R^, a *cos* site for DNA packing in lambda phages. This allows the cloning of very long DNA fragments (up to 50 kb) by ligation and packing into empty lambda phages. The packed DNA is then used to infect *E. coli* host strains, resulting in the construction of genomic or metagenomic DNA libraries.

Plasmid vectors are usually chosen to generate small‐insert metagenomic libraries (< 10 kb average insert size), becoming an appropriate tool to identify single gene products encoded by small DNA sequences, such as enzymes and antibiotic resistance genes (Guazzaroni *et al*., 2010). Regulating the expression level of cloned genes is possible by using inducible promoters upstream of the DNA insertion site or by choosing a suitable plasmid copy number, avoiding high expression rates of toxic genes or inclusion body formation of target proteins, and low expression rates that could prevent detection in functional screenings (Hudson, 2001). Large‐insert metagenomic libraries are usually generated using cosmid or fosmid vectors and provide a more efficient method to identify complete operons and biosynthetic clusters of genes. Cosmids are vectors capable of carrying DNA inserts of about 30–40 kb in size that contain the *cos* site of λ phage for packing inserted metagenomic DNA in λ phages (Hohn and Collins, 1980). This type of vector can replicate in suitable hosts since it carries a proper origin of replication, but the lack of a copy number control mechanism usually decreases cosmid stability (Haley, 1988; Cheng *et al*., 2017). Fosmid vectors are similar to cosmids, but they use an *E. coli* F‐factor origin of replication, making the fosmid copy number highly regulated to 1 or 2 copies in the cell and replication is restricted to *E. coli* (Kim *et al*., 1992; Fig. 6). This fact could avoid gene toxicity interference in metagenomic tests; also, fosmids are capable of carrying large‐insert DNA sizes of about 40–50 kb (Santana‐pereira and Liles, 2017). One example of a regularly used fosmid is the pCC1FOS (Epicentre, Madison, Wisconsin), a commercial cloning vector that allows copy number to be controlled through the CopyControl Cloning System. The pCC1FOS fosmid was first introduced in metagenomic studies in 2004 in a proof‐of‐concept report using a (meta)genomic library from *Collimonas fungivorans* (Leveau *et al*., 2004). Yet, for even larger fragments, bacterial artificial chromosomes (BACs) are used. These vectors are modified plasmids that contain an origin of replication derived from the *E. coli* F‐factor and can stably maintain and replicate inserts ranging from 100 kb to 220 kb, as well as inserts of more than 300 kb, and are usually used in *E. coli* (Shizuya *et al*., 1992; Beja *et al*., 2000).

### Functional metagenomics: using broad‐host‐range vectors to enhance the probability of identifying target genes

An alternative to overcoming the limitations of metagenomic approaches and to enhance the discovery of new biocatalysts and molecules of interest is the use of alternative host organisms (besides *E. coli*) to perform the functional screening tests. For this, shuttle vectors and broad‐host‐range vectors have been mostly used. This type of vector (see Fig. 2) has been largely used in functional metagenomics, increasing the efficacy of identifying target activities (Table 2). Vectors used in metagenomic screening in alternative hosts contain a single broad‐host origin *oriV* or a multiple *oriV,* which permit its replication in *E. coli* and other hosts, or integrative‐based systems that allow integration of the metagenomic DNA into the chromosome of the screening host (Cheng *et al*., 2017).

**Table 2:** Multiple host‐vector systems for metagenomic DNA cloning and functional screening

Library vector	Origin type/Vector type	Vector size	Relevant characteristics	Average insert size	Environment	Screening hosts	Library size (Mb or Gb)/Number of clones	Gene/function target	References
pLF61	Shuttle/plasmid	5.4 kb	PGK promoter; URA3 gene; origins from yeast 2 μm plasmid *E. col*i vector pUC19	1–3 kb	Soil eukaryote organisms (metatranscriptome)	*S. cerevisiae* W303	1.75 × 10^6^	Oligopeptide transporters	Damon *et al*. (2011)
pMM436	Shuttle/cosmid	Not shown	ColE1 origin for *E. coli* and integrative *attB* for *S. lividans;* PacI restriction site flanking attB integration site for recovering metagenomic DNA	30–40 kb	Libraries I and II different soil samples	*S. lividans* ∆act ∆red	I) 367.5 Mb/10 500 II) 2485 Mb/71 000	I) Haemolytic activity II) Pigmentation producing	McMahon *et al*. (2012)
pCT3FK	Shuttle/fosmid	11.6 kb	RK2 origin, *pyr* (chromosomal surroundings of the *pyr* locus allow homologous recombination in *T. thermophilus*), KmR	35–40 kb	Water, sediment, biofilm from hot springs	*T. thermophilus* BL03, E. coli EPI300	~8000	Esterase‐active enzymes	Leis *et al*. (2015)
pCT3FK	Shuttle/fosmid	11.6 kb	RK2 origin, *pyr* (chromosomal surroundings of the *pyr* locus were inserted in the vector to allow homologous recombination in *T. thermophilus*), KmR	50 kb	*S. thermophila* (genomic library)	*E. coli* K12 and *T. thermophilus* HB27	2.9 Mb/192	Xylanase activity	Angelov *et al*. (2009)
pLAFR3	Broad‐host‐range/cosmid	22 kb	pLAFR1 with *HaeII* fragment (MCS and a‐complementation for ß‐galactosidase activity), TcR, cos site, RK2 origin	25 kb	Wastewater treatment plant anaerobic sludge digestor	*E. coli* and *R. leguminosarum* bv. Viciae	2750 Mb/110 000	Dehydrogenase genes	Wexler *et al*. (2005)
pGNS‐BAC‐1	Shuttle/BAC	11.9 kb	Two origins of replication (i.e. F and RK2), *oriV* origin, arabinose‐inducible plasmid copy number, *CmR*.	80 kb	Forest soil	Clones from *E. coli* were electroporated into *S. marcescens*,* V. cholerae*,* E. nimipressuralis*	Not shown	Not shown	Kakirde *et al*. (2011)
pOS700I	Shuttle/cosmid	Not shown	AmpR, *cos* sequence, pOS700I integrative in *S. lividans* via *attB* site and *int* gene from the *Streptomyces* integrative element pSAM2, allowing site‐specific integration of pOS700I in many *Streptomyces* species	50 kb	Soil from an arable field	*E. coli*/*S. lividans* (only to check phenotypes found in *E. coli)*	250 Mb/5000	Polyketide synthase genes	Courtois *et al*. (2003)
pWEB436	Shuttle/cosmid	Not shown	ColE1 origin, *cos* site, AmpR, ΦC31 integration system for integration in *Streptomyces*, ApraR, oriT from the lncP plasmid	40–50 kb	Texas desert soil	*Streptomyces albus*	1.5 million	Polyketide synthase genes	Iqbal *et al*. (2016)
pSrpsL14	Shuttle/BAC	Not shown	ColE1 origin, pSG5 origin of *Streptomyces*; AmpR (*E. coli);* ThioR (*Streptomyces*); GmR (*E. coli and Streptomyces*)	50–100 kb	Soil	*S. lividans* ∆act ∆red, *P. putida* MBD1	Not shown	Antibacterial and antifungal activities	Martinez *et al*. (2004)
pKS13S	Broad‐host‐range/cosmid	21.7 kb	RK2, TcR, *cos* site, RK2 *oriT*.	25 kb	Oil‐contaminated soil	*P. putida* G7K2 and KTSK2	294 Mb/24 000	Naphthalene‐catabolic genes	Ono *et al*. (2007)
pJWC1	Broad‐host‐range/cosmid	14 kb	*trfA*, RK2 *oriT*, RK2, TcR, ApR, RK2 stability locus (0.8 kb).	High‐molecular weight	Deciduous forest topsoil, creek bed mud/sediment and sand/clay‐covered cold desert soil	*Agrobacterium tumefaciens* LBA4404, *Burkholderia graminis* C4D1M, *Caulobacter vibrioides* CB15, *Escherichia coli* EC100, *Pseudomonas putida* KT2440, *Ralstonia metallidurans* CH34	750 000	Pigmentation producing and antibacterial activity	Craig *et al*. (2010)
pJWC1	Broad‐host‐range/cosmid	14 kb	*trfA*,* oriT* (RK2 origin of transfer), RK2, TcR, ApR, RK2 stability locus (0.8 kb)	High‐molecular weight	Soil	*Ralstonia metallidurans* CH34	575 000	Metabolites from pigmented compounds and antibacterial	Craig *et al*. (2010)
pKS13S	Broad‐host‐range/cosmid	21.7 kb	RK2 origin, TcR, cos site, RK2 *oriT*	25 kb	Artificially polluted soil (with biphenyl, phenanthrene, carbazole and 3‐chlorobenzoate)	*P. putida* KTSK2 and *P. putida* G7NAD2	5.2 Gb/208 000	Oxygenase genes	Nagayama *et al*. (2015)
pJC8	Broad‐host‐range/cosmid	13 kb	TcR, GmR, Rk2, *oriT*, Gateway attL sites used for recombination transfer of cosmid inserts into other destination vectors	33 kb	Soil from the wheat field	*P. putida* PpUW2 (PHA‐)	362 Gb/9 × 10^6^	Polyhydroxy‐alkanoate synthases genes	Cheng and Charles (2016)
pRS44	Broad‐host‐range/fosmid and BAC	10.3 kb	CmR, KmR, stabilization element parDE (from RK2), *oriT*,* Lac* system for blue/white screening, *ori2* origin (from the F plasmid) *oriV* from RK2	35 kb	Marine sediment	*Pseudomonas fluorescens* and *Xanthomonas campestris*	20 000	Not shown	Aakvik *et al*. (2009)

A variety of studies have found that using diverse hosts when screening metagenomics libraries can increase the discovery rate of active clones (Table 2). Metagenomic libraries are often directly constructed in *E. coli* due to the higher number of transformants obtained when compared with the low rate of transformants achieved in other alternative hosts. Thus, to perform screenings in alternative hosts, a common method is to use shuttle or broad‐host‐range vectors for library construction in *E. coli* and then to transfer and screening these libraries in other host organisms. For instance, Damon *et al*. (2011) used the pFL61 yeast‐*E. coli* plasmid shuttle vector to construct a small‐insert library from soil eukaryote DNA using a metatranscriptomic approach. Performing functional complementation of a yeast mutant defective in a di/tripeptide, they identified a novel family of oligopeptide transporters expressed by fungi. Also, the pMycoFos fosmid shuttle vector has been used to allow transfer of a butane‐oxidizing strain *Nocardioides* CF8 DNA from *E. coli* EPI300 to *Mycobacterium* spp (Ly *et al*., 2011). In addition, McMahon *et al*. (2012) developed a shuttle cosmid vector for *E. coli* and *Streptomyces lividans* and used an optimized *S. lividans* strain for screening. In another study, Martinez *et al*. (2004) used a pSrps14 shuttle BAC vector and showed different functions among *E. coli*,* P. putida* and *S. lividans* expressing heterologous metagenomic genes.

On the other hand, several studies using broad‐host‐range vectors also have been reported in metagenomics screenings. For instance, Craig *et al*. (2010) described the construction of a cosmid library from soil samples in a broad‐host‐range vector (pJWC1), which was screened for antibacterial activity, altered pigmentation and altered colony morphology in six different Proteobacteria*: A. tumefaciens, Burkholderia graminis, Caulobacter vibrioides, E. coli, P. putida* and *Ralstonia metallidurans*. The screenings in *E. coli* identified two clones displaying antibiosis activity, but no clones displayed either pigmentation or morphology alterations, and the screenings in other hosts identifying a number of other target characteristics. This indicates that the same metagenomic library can yield different results depending on the expression host used (Craig *et al*., 2010).

Moreover, Nagayama *et al*. (2015) constructed a metagenomic library from artificially polluted soil samples using a broad‐host‐range vector (pKS13S) based on the RK2 origin of replication, showing different success rate when analysed the library in *P. putida*,* E. coli* and *B. multivorans*. Leis *et al*. (2015) used a *T. thermophilus/E. coli* shuttle fosmid vector (pCT3FK; Angelov *et al*., 2009) to generate a large‐insert metagenomic library and performed screenings for lipolytic activities in *E. coli* and *T. thermophilus* HB2 (using a multiple clean deletion mutant *T. thermophilus)* which lacks several characterized extracellular and putative esterase‐encoding genes. They found two thermostable a/b‐fold hydrolase enzymes with high amino acid sequence similarity to already characterized enzymes in *E. coli* screening. In contrast, they found six fosmids that conferred lipolytic activities to *T. thermophilus*.

Additionally, in the last years, diverse efforts have been made to produce new vectors displaying relevant characteristics, and synthetic biology has become a powerful tool for this (Guazzaroni *et al*., 2015; Alves *et al*., 2017b). For instance, Bryksin and Matsumura (2010) described an engineered broad‐host‐range origin of replication (pWV01 RCR) used to create the high copy number vector pBAV1K‐T5, which can replicate in different Gram‐negative and Gram‐positive bacterial species. In another study, Terrón‐González *et al*. (2013) developed vectors and specialized *E. coli* strains as improved metagenomic DNA heterologous expression systems, which was based on the T7 RNA‐polymerase and the lambda phage transcription anti‐termination protein N (Terrón‐González *et al*., 2013). However, the approaches developed are limited to *E. coli* as a host. Another alternative to overcome the low expression of heterologous genes in functional metagenomics was proposed by Gaida *et al*. (2015). Authors created *E. coli* strains expressing heterologous sigma factors that are able to recognize heterologous promoters from metagenomic and genomic DNA libraries. The study showed that RpoD from *Lactobacillus plantarum* can initiate transcription from all sources of tested DNA. Moreover, the use of modular vectors, such as the pSEVA vectors listed above (Silva‐Rocha *et al*., 2013), confers diverse benefits for constructing metagenomic libraries. Yet, besides all the advantageous characteristics already mentioned, these vectors only allow the cloning of small metagenomic DNA fragments (up to 10 kb) and are not suitable for constructing large‐insert DNA libraries, which are essential for functional recovery of complete biosynthetic pathways involved in producing bioactive compounds (as in the case of non‐ribosomal peptide synthetases, polyketide synthases and terpene synthases genes, among others). Therefore, since metagenomics is a rising field, innovations in tools to facilitate manipulation and promote the discovery of functional genes in environmental samples are still needed.

## Challenges and perspectives in vector design

**Figure 7:**
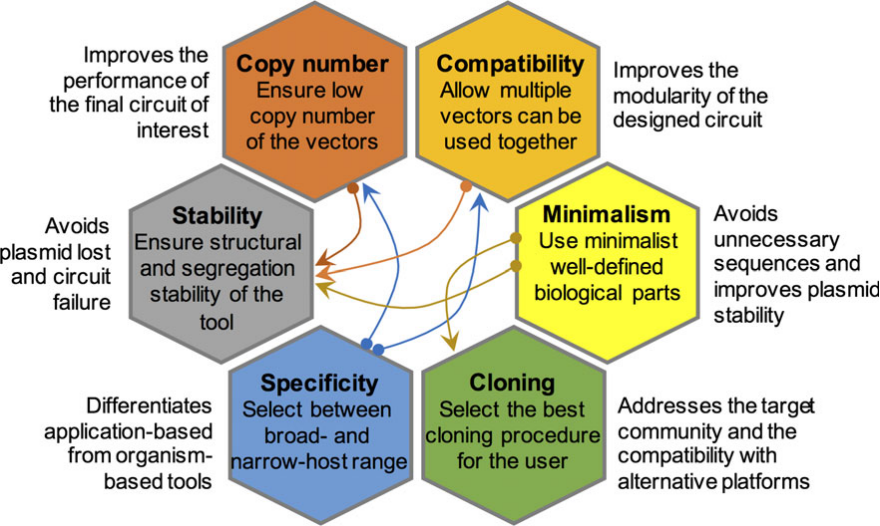
Critical features to consider for efficient vector engineering. In this schematic illustration, outside hexagons represent the main impact of each step on the effectiveness of the tool. Arrows connecting hexagons indicate which features significantly impact each other.

When considering the tremendous advances in vector engineering, it becomes evident that even the sophisticated set of tools constructed so far cannot solve all the bottlenecks existing in the field. In this section, we present some of the challenges that need to be taken into account when designing the next generation of modular genetic tools (Fig. 7).

### Copy number control

While plasmid vectors represent an easy way to insert and test synthetic circuits in microorganisms, they usually replicate, generating more copies per cell and more chromosomes. Indeed, it has been increasingly recognized that circuits implemented in low copy exhibit enhanced performance compared to those placed in multicopy (Lee *et al*., 2016). Since vector engineering usually requires the modification of the *ori* of replication or of its surrounding area, special care must be taken to determine if those changes modify the copy number of the final vector. In this context, some very elegant approaches using fluorescent activated cell sorting (FACS) and digital PCR have been established to assay variations and plasmid copy number (Jahn *et al*., 2016), and variations of these methods are highly recommended in new vector design projects.

### Plasmid incompatibility

The use of multiple plasmids to implement complex synthetic circuits is a very attractive approach since it allows the optimization of the whole system in a modular way. However, while bacterial plasmids use diverse mechanisms for autonomous DNA replication, some of these require the same host machinery. As a result, many origins of replications belong to the same incompatibility group, which means that they cannot be stably maintained simultaneously in the same host (Novick, 1987). Therefore, it is imperative to consider plasmid incompatibility groups when designing novel genetic tools. Alternatively, it would be possible to create orthogonal origins of replication through engineering *Rep* proteins, but this type of approach has not been reported yet.

### Plasmid structural and segregation stability

Two fundamental features of plasmid biology that are virtually neglected in recent engineering approaches are the structural and segregation stability of the vectors. These features were intensively investigated in the 1980s, as researchers reported that many natural plasmids presented spontaneous loss during cell division (segregation instability) or displayed profound rearrangements in their structures and loss of DNA segments (structural stability; Maschke *et al*., 1992). The former process would occur due to defects during replication of the plasmid DNA and to the absence of the specific segment of the plasmid responsible for efficient partition of vectors to the daughter cells (Nordström and Austin, 1989). The latter, though, could be due to many reasons, from an excess of homologous sequences in the plasmid backbone to the high‐level expression of toxic genes in the plasmid, which may result in a fitness advantage for those cells that harbour mutant versions of the systems with the deleted gene (Ehrlich *et al*., 1991). When designing new synthetic vectors, it is important to ensure that the final tool is stable enough to guarantee the performance of the final circuit of interest.

### Use of minimalist, fully characterized parts

The use of minimalist DNA fragments is also a good practice in vector design to allow the final tool to be as minimal as possible. This is manageable for bacterial plasmids but is not trivial for vectors designed for yeast and filamentous fungi, for example, where there is a lack of consistent information regarding minimal regulatory elements. For those cases, the characterization of the individual biological parts is crucial for the use of the appropriate fragments and to ensure the reliability of the final tool.

### Universal versus case‐specific platforms

The dualism between universality vs. specificity is best represented by broad‐ and narrow‐host‐range vectors. This is particularly important as the field of synthetic biology moves into real applications where non‐model organisms may be required (Bassalo *et al*., 2016). While designing narrow‐host‐range platforms allows the construction of tools that meet the specific requirements of the hosts, broad‐host‐range vectors allow the user to easily switch the host without requiring the reconstruction of the circuit of interest. Yet, the way each host interacts with the genetic elements of the tools can vary drastically, which could impair the functioning of, for example, some regulatory elements of the vector. In that sense, the new generation of universal tools should consider the use of orthogonal elements to ensure the efficient recognition of such regulatory elements in the targeted hosts. Examples of this have been the recent engineering of synthetic promoters efficiently recognized by Gram‐negative and Gram‐positive bacteria, as well as by yeast (Yang *et al*., 2018).

### Selection of appropriate circuit‐cloning methods

When designing novel genetic tools, it is imperative to consider the final target community and their preferences. While the initial progress in plasmid engineering was built upon the use of restriction enzymes and DNA ligase, restriction‐free methods are becoming more and more popular in the synthetic biology community (Ellis *et al*., 2011). Moreover, the continuous decline of DNA synthesis costs will certainly lead to a future where synthetic biology projects will rely on complex circuits encoded in several kb of DNA constructed by *de novo* synthesis. Looking further forward, an immediate need for a valuable genetic tool is to reach virtually any user and be based in the most straightforward cloning procedures.

## Concluding remarks

Bacteria and fungi are multifaceted organisms containing several layers of complexity. Here, we have briefly reviewed the major events that have shaped the field of vector design for all those microorganisms over the past decades. In summary, the two major requirements for exceptional vectors are versatility and modularity. Undoubtedly, most tools were built based on the model organisms from each Kingdom: *E. coli* and *S. cerevisiae*. However, we realize now that those technologies are on an inevitable path to expand to other microorganisms due to their enormous importance in health and industry. Broad‐host‐range vectors and shuttle vectors are part of that solution. Ideally, the same tool should work for both the model and the other target organism. Versatility would benefit not only fundamental science but would also help the search for new metagenomic products. Yet, modularity, as stated several times throughout the review, is the ultimate stage we must reach to enter the new era of synthetic biology‐based vectors. To achieve that, we need a complete and extensive characterization and standardization of all biological parts, either inside or outside biological systems. Perhaps we will not find an ‘one vector for them all’ solution, where a single platform can be used for any organism of interest. Therefore, we anticipate a situation where basic design rules are generated in as many model organisms as possible and then are applied to new organisms using DNA synthesis technologies that are continuously decreasing in cost.
